# The Prediction and Evaluation of Surface Quality during the Milling of Blade-Root Grooves Based on a Long Short-Term Memory Network and Signal Fusion

**DOI:** 10.3390/s24155055

**Published:** 2024-08-05

**Authors:** Jing Ni, Kai Chen, Zhen Meng, Zuji Li, Ruizhi Li, Weiguang Liu

**Affiliations:** School of Mechanical Engineering, Hangzhou Dianzi University, Hangzhou 310018, China; nj2000@hdu.edu.cn (J.N.); 222010099@hdu.edu.cn (K.C.); 222010116@hdu.edu.cn (Z.L.); 202243010062@hdu.edu.cn (R.L.); 232010128@hdu.edu.cn (W.L.)

**Keywords:** surface quality, 21Cr13, correlation analysis, long short-term memory network, signal fusion, blade-root groove

## Abstract

The surface quality of milled blade-root grooves in industrial turbine blades significantly influences their mechanical properties. The surface texture reveals the interaction between the tool and the workpiece during the machining process, which plays a key role in determining the surface quality. In addition, there is a significant correlation between acoustic vibration signals and surface texture features. However, current research on surface quality is still relatively limited, and most considers only a single signal. In this paper, 160 sets of industrial field data were collected by multiple sensors to study the surface quality of a blade-root groove. A surface texture feature prediction method based on acoustic vibration signal fusion is proposed to evaluate the surface quality. Fast Fourier transform (FFT) is used to process the signal, and the clean and smooth features are extracted by combining wavelet denoising and multivariate smoothing denoising. At the same time, based on the gray-level co-occurrence matrix, the surface texture image features of different angles of the blade-root groove are extracted to describe the texture features. The fused acoustic vibration signal features are input, and the texture features are output to establish a texture feature prediction model. After predicting the texture features, the surface quality is evaluated by setting a threshold value. The threshold is selected based on all sample data, and the final judgment accuracy is 90%.

## 1. Introduction

For the operation of industrial steam turbines, steam turbine blades are one of the core components; as a key medium for energy conversion, they play an indispensable role in steam turbines, and the quality of steam turbine blades will directly affect the overall quality of power equipment [1]. During the operation of industrial steam turbines, the blades are subjected to the impacts of high temperature, high pressure, and high-speed gas, which leads to the need for the blade-root groove to be perfectly assembled with the rotor to improve the life of the blade-root groove. Therefore, research on the milling performance of the blade-root groove is particularly important. The surface quality resulting from the milling of blade-root grooves has an important influence on their performance. The quality of the surface texture is directly related to the functional performance of the blade-root groove and the reliability of the workpiece [2]. By controlling the quality and characteristics of the surface texture, the cutting forces can be reduced, the surface quality can be improved, and the tool life can be extended, resulting in efficient, stable, reliable blade-root and groove milling.

Regarding surface quality, Otsuki et al. [3] studied the relationship between the brightness and roughness of the machined surface, and the surface quality was evaluated by the method of surface luminance imaging. Deng et al. [4] studied the influence of different areas of the optical surface on the surface quality, and an optical surface quality evaluation method considering local tolerances was proposed. Yu et al. [5] studied the effects of tool wear and machining surface hardness on surface roughness, and it was found that surface hardness had the greatest influence on surface roughness, and a surface roughness prediction model was established. Zhao et al. [6] carried out ultrasonic vibration-assisted grinding experiments, and a surface roughness prediction model was established based on motion mechanics analysis to obtain a better surface quality.

Most existing research focuses on the relationship between surface images and surface roughness, predicting and evaluating surface quality through the development of prediction models. However, the processed surface images are often significantly affected by external conditions, such as light intensity, chips, and cutting fluid, which will seriously interfere with the acquisition and analysis of surface images. These external interference factors pose significant challenges for researchers to evaluate and predict surface quality. Therefore, it is necessary to propose an effective method to achieve accurate evaluation and prediction of the surface quality of blade-root grooves, to ensure that reliable evaluation results can still be obtained under different external conditions.

Surface texture is the essence of the surface [7]. Here are some methods for extracting texture features from images [8,9,10]. Although the aforementioned methods are effective, they require obtaining texture images from the cleaned surface. Cutting vibration has a significant effect on the final surface topography [11]. On this basis, the vibration signals obtained during processing can be used to indirectly evaluate the surface texture [12]. In addition, the sound signals generated in the milling process also reflect the surface texture characteristics to a certain extent. Therefore, this paper aims to accomplish the prediction and evaluation of surface quality based on vibration signals and sound signals.

Based on the correlation between machining signals and surface texture features, the surface texture prediction model of the workpiece is established. Some scholars have applied physical modeling methods as well as modern artificial intelligence methods such as BP neural, support vector machines, and the grayscale covariance matrix to establish a prediction model for surface quality [13,14]. Liu et al. [15] extracted the multi-scale characteristics of the sensor signal using a residual network, and the prediction of the tool wear value was realized. Gao et al. [16] denoised data by wavelet denoising and phase space reconstruction, and the obtained reconstructed phase space was used as the input for an LSTM neural network. Miao et al. [17] proposed a prediction model for circumferential milling of the machined surface that considers the runout of the tool and the dynamic displacement of the workpiece. Andrews et al. [18] collected the vibration signals during the cutting process and extracted the features that are useful for the surface condition, and they were able to predict the surface roughness. By extracting the theoretical values of AE signals and surface roughness as inputs for the support vector regression model, Tang et al. [19] were able to achieve the prediction of surface roughness. However, surface texture feature prediction is essentially time-series modeling. Compared to many other methods, LSTM neural networks possess a unique cell structure that enables them to learn long-term dependencies, making them suitable for the classification, processing, and prediction of time-series data [20]. Therefore, in this paper, the LSTM neural network is utilized to establish a prediction model for the surface texture features of leaf root grooves.

Firstly, the milling experiment of martensitic stainless steel (21Cr13) was carried out to obtain the sound signal, vibration signal, and workpiece surface image during the machining process. Then the collected signals were preprocessed, and the blade-root grooves surface texture features were extracted by using the gray-level co-occurrence matrix. Correlation analysis was performed on the processed signal and texture features. Then, a prediction model of the leaf root surface texture features was established by using a long short-term memory (LSTM) neural network and an acoustic vibration signal as input. Finally, the surface quality is evaluated by the predicted surface texture features.

## 2. Experimental Design

### 2.1. Experimental Platform Construction Scheme

The material used in this experiment is martensitic stainless steel (21Cr13), which mainly consists of four chemical components: nickel, carbon, chromium, and silicon. Martensitic stainless steel has a high thermal strength, good vibration damping, and organizational stability, and its mechanical and physical properties are shown in Table 1. The tool used is a three-face end mill, and its material is carbide. Tungsten carbide tools have a good hardness and the ability to maintain a sharp cutting edge at high temperatures, making them ideal for machining tough materials such as martensitic stainless steel. The combination of the selected workpiece material and the cutting tool ensures that the machining characteristics and surface quality results under various milling conditions can be effectively investigated.

**Figure 1:**
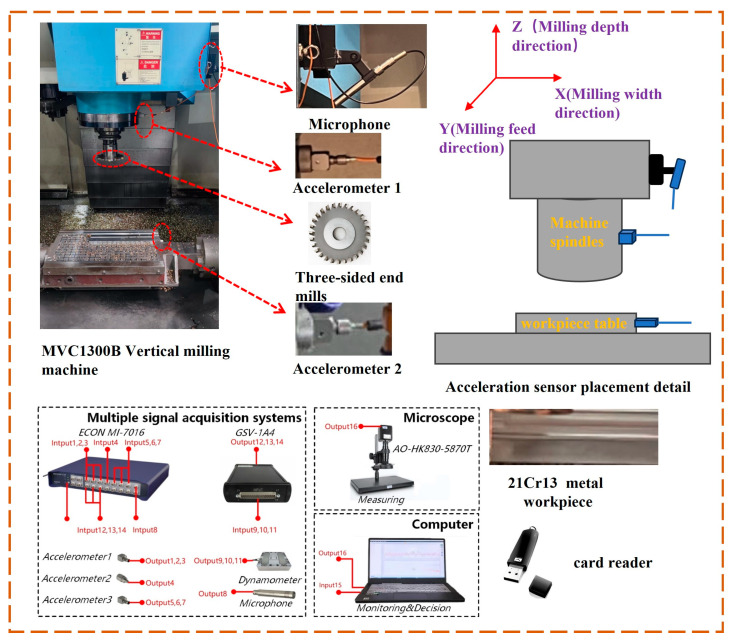
Data acquisition platform.

The experiment was carried out on the MVC1300B Ouma vertical milling machine, and the three-way acceleration sensors were arranged on the main axis of the machine as well as the workpiece table during the experiment, and the microphone sensors were arranged on the main axis of the machine as shown in Figure 1. For the milling of the entire blade-root groove, two operations were performed. During the first milling, the worktable moved at a speed of 240 mm/s; during the second milling, the worktable moved at a speed of 270 mm/s. In total, 160 experimental sets were collected. The spindle speed of 350 rpm, the feed per tooth of 0.078 mm, and the cutting speed of 137.7 mm/min were selected from the machining manual and manual experience, and the cutting time was increased by 0.5 min between two adjacent groups. The equipment used to collect the data were a data collector, three acceleration sensors, a microphone sensor, an electron microscope, a laptop computer, a monitor, and an acquisition card. Table 2 shows the information on the tool material, workpiece material, and process parameters.

### 2.2. Experimental Data Acquisition

During the experimental process, a turbine blade with dimensions of 450 mm in length, 100 mm in width, and 50 mm in height was securely clamped using a fixture. A three-face end milling cutter performed two milling operations on the blade-root grooves, producing a groove measuring 450 mm in length, 30 mm in width, and 25 mm in depth. The table feed rates were set at 240 mm/s for the first cutter and 270 mm/s for the second cutter, with a spindle speed of 350 rpm. Vibration signals in the X, Y, and Z directions of both the machine tool spindle and the workpiece table were collected using a data acquisition instrument (ECON MI-7017, Hangzhou YiHeng Technology Co., Ltd., Hangzhou, China) during the initial milling and at the end of the second milling. Additionally, sound signals were recorded throughout the machining process. If no signal fluctuations were detected, the sensor was repositioned to ensure signal consistency. The sampling frequency was set to 5120 Hz, with an analyzed spectral line of 1600 Hz, 4096 sampling points, and an analyzed bandwidth of 2000 Hz. Data collected from each experiment were saved in DAR format for easy access in later stages.

**Figure 2:**
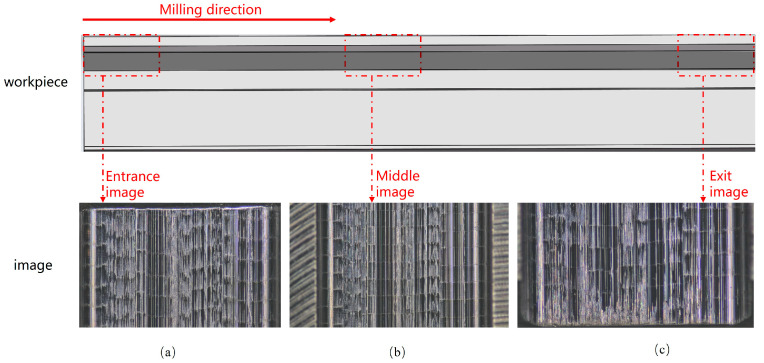
(**a**–**c**) represent the images of the entrance, middle, and exit sections of the workpiece after milling.

Upon the completion of each experimental set, the surface images of the machined blade-root grooves were acquired using an electron microscope (model AO-HK830-5870T), as illustrated in Figure 2. A lens with a magnification of 20 was selected for the size of the workpiece and the height of the electron microscope. To enhance data quantity and accuracy, images were taken at the entrance, center, and exit of the processed workpiece, and sequentially stored on a capture card. During the milling experiments, a total of 160 sets of experimental data were collected, and 480 images of the workpiece surface after milling were captured.

## 3. Feature Extraction and Correlation Analysis

**Figure 3:**
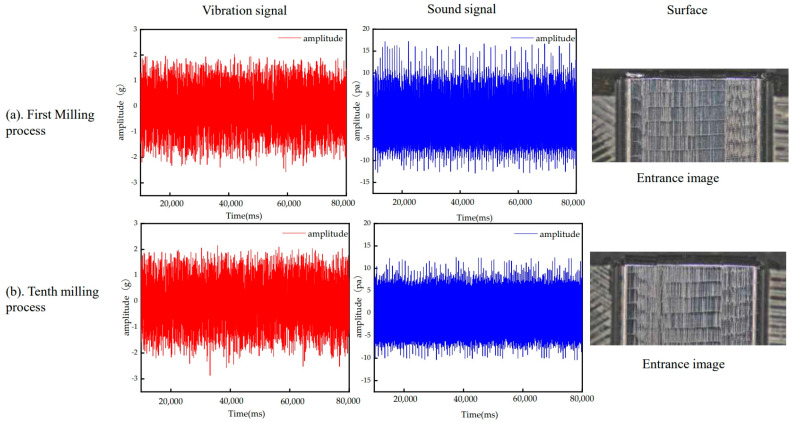
(**a**) is the signal and workpiece surface in the first machining case. (**b**) is the signal and workpiece surface in the tenth machining case.

In the milling process, the surface quality of the workpiece can be assessed and judged by analyzing the signals related to workpiece machining, and vibration signals are a commonly used signal for assessing the surface quality of the workpiece during machining [21]. Additionally, in some specific machining processes, acoustic signals can also be used to assess the surface quality of the workpiece. For example, in metal machining, the surface finish and flatness can be determined by analyzing the sound signals generated during the machining process [22]. As shown in Figure 3, the data collected in this paper show that as the vibration signal becomes larger and the sound signal becomes smaller, the surface quality of the workpiece processed is worse. According to the results of 160 sets of experiments, it can be preliminarily judged that there is a certain relationship between the signal features of the machining process and the surface quality.

### 3.1. Signal Feature Extraction

**Figure 4:**
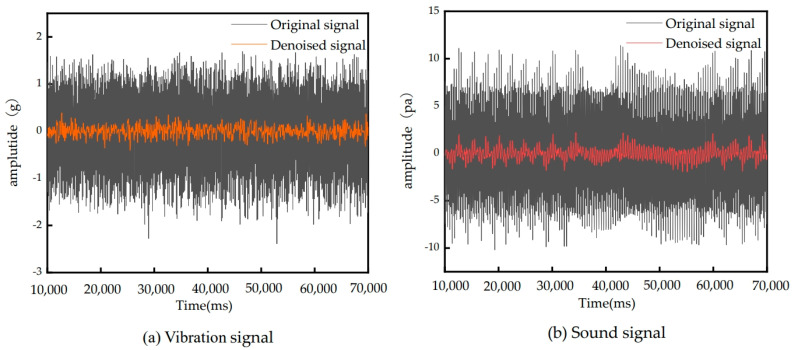
Signals after wavelet denoising processing.

Firstly, the collected sound signal and vibration signal data are verified to see whether the basic data are in line with the actual situation, and redundant data and error data are deleted. The collected vibration signal and sound signal are filtered by a fast Fourier transform. Finally, the noise component in the signal is effectively reduced by wavelet denoising to improve the quality of the signal and make the analysis and processing algorithm more accurate and reliable. According to the artificial experience of the laboratory, the parameters of the fast Fourier transform (FFT) are set as follows: the sampling frequency is 5120 Hz, the number of sampling points is 4096, and the window type is Hanning Window. In addition, the Haar wavelet function is used for denoising, and the threshold is set to 50% each time. Correct processing of the signal is essential for building an accurate and reliable prediction model [23]. The processing results of the vibration and sound signals collected from the first milling process are shown in Figure 4.

A time–frequency analysis of the vibration and sound signals was conducted to obtain the maximum amplitude, minimum amplitude, average amplitude, root-mean-square error, crest factor, skewness, and other characteristics. In order to reduce the variability of the extracted signal features, this study employs polynomial smoothing for denoising, thereby obtaining more robust features [24,25]. Polynomial smoothing filtering can make the signal smoother and the features obtained better.

**Figure 5:**
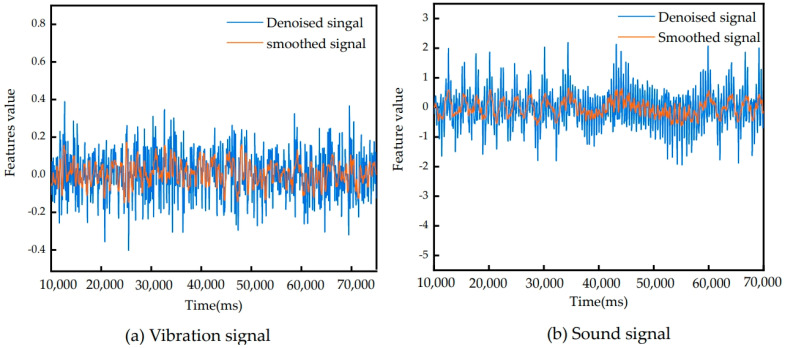
Polynomial smoothing window processing signal.

The setting of the window length has a great influence on the result of the smoothed features [26]. In this paper, when using polynomial smoothing, the number of window points is set to 50, Polynomial smoothing window denoising is performed on the above wavelet denoised signal data, as shown in Figure 5.

Smoother and cleaner feature representations are obtained by a combination of wavelet denoising and polynomial smoothing denoising. Then, correlation analysis is performed, and finally, the features with a higher correlation are used as inputs to the prediction model.

### 3.2. Surface Texture Image Feature Extraction

The gray-level co-occurrence matrix (GLCM) is a significant statistical method for texture feature description [27], based on the grayscale relationships between pixels.

Before utilizing GLCM, histogram normalization is performed on each original image to minimize the impact of uneven illumination on grayscale values [28]. GLCM is a list of the frequency of occurrence of different combinations of gray levels in an image [29]. Formally, it can be shown:

(1) $$G{\left(i,j\right)}_{d,\theta }=\left\{\left(p_{1},p_{2}\right)|I\left(p_{1}\right)=i,I\left(p_{2}\right)=j\right\}$$

where the variables i and j are converted to gray levels. $p_{1}$ and$\;p_{2}$ are two image pixels and$\;p_{2}$ is situated at angle θ, distance d with respect to $p_{1}$. Based on this definition, five kinds of image texture features can be obtained from GLCM: energy (ASM), contrast (CON), entropy (ENT), uniformity (IDM), and correlation (COR).

The surface texture state is evaluated based on the overall uniformity and smoothness in all directions, not in one direction. Then, the texture feature values of different directions are combined to form a system indicator. The grayscale symbiosis matrix was used to extract the average of the surface texture features in the four directions of the workpiece, including 0, 45, 90, and 135, are calculated. The texture image feature vector consists of the average of all these features.

V = [*AVE_ASM, AVE_CON, AVE_ENT, AVE_IDM, AVE_COR*] (2)

where *AVE_ASM*, *AVE_CON*, *AVE_ENT*, *AVE_IDM,* and *AVE_COR* are, respectively, average. Due to the lack of quantitative surface quality assessment standards [30], it is challenging to determine whether the surface quality is acceptable. The surface image features are obtained by image analysis and the gray-level co-occurrence matrix method for tool wear classification [31]. The root-mean-square value and energy of the machined surface can be extracted by wavelet transform to evaluate the surface quality [32]. Here, we use a relative reference sample to identify textures with better smoothness and uniformity. Assuming V0 = [${AVE\_ASM}_{o}$, ${AVE\_CON}_{o}$, ${AVE\_ENT}_{o}$, ${AVE\_IDM}_{o}$, ${AVE\_COR}_{o}$] is the threshold for an acceptable texture,

$$AVE\_ASM\;<\;{AVE\_ASM}_{o}$$

$$AVE\_CON\;>\;{AVE\_CON}_{o}$$

$$AVE\_ENT\;<\;{AVE\_ENT}_{o}$$

$$AVE\_IDM\;>\;{AVE\_IDM}_{o}$$

$$AVE\_COR\;>\;{AVE\_COR}_{o}$$

**Figure 6:**
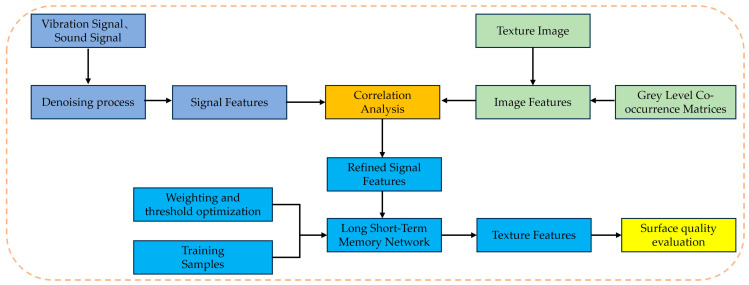
Surface quality evaluation process.

The surface quality assessment process is illustrated in Figure 6 and is based on all the criteria above. If the surface texture feature meets all of the above thresholds, the workpiece surface quality is acceptable, otherwise it is unacceptable.

### 3.3. Correlation Analysis

Before utilizing vibration signal features and sound signal features to predict image texture features, it is necessary to conduct a correlation analysis. Assume that *y*(*t*) and *z*(*t*) represent a surface image texture feature and a vibration signal feature, respectively. The correlation coefficient [33] between them is noted by $R_{yz}(\tau )$ as

(3) $$R_{yz}(\sigma )=\int_{0}^{T}y(t)z(t+\sigma )dt$$

Similarly, the correlation analysis of sound signal features shows that the sound signal features are also highly correlated with the surface texture features of the image.

The correlation analysis of the collected experimental data was carried out by the above method. The analysis identifies the vibration signal features with the strongest correlation with the image texture features, as well as the sound signal features, as detailed in Table 3. Based on the above discussion, it can be found that the image texture features have a large correlation with the average amplitude (AVE_IMF), maximum amplitude (MAX_IMF), skewness, and root-mean-square (RMS) value.

The above correlation analysis can be used to know the mapping relationship between texture features and sensitive signal features.

## 4. The Evaluation of Surface Quality Based on LSTM and Signal Fusion

### 4.1. Model Building

During the milling process, spindle vibration and tool wear inevitably impact the surface texture of the leaf root groove. To evaluate surface quality and optimize subsequent process parameters, this paper proposes a surface quality evaluation method. The method primarily utilizes the collected spindle vibration signals and acoustic signals during the machining process as inputs, and the surface texture feature values as outputs, to construct a surface texture feature prediction model. To improve the accuracy of the predictions, correlation analysis is performed before inputting the signal features, and weights are assigned based on the results of this analysis. Finally, the surface quality of the workpiece is evaluated based on the predicted surface texture features. This method allows for a timely evaluation of surface quality without considering the effects of lighting on captured images or concerns about cutting fluid and chips obstructing the workpiece.

In the LSTM model, regular neurons are replaced by memory units. In the memory unit, the structures that are removed or added by the management unit are called thresholds. There are three types of thresholds: forget gates, input gates, and output gates. Thresholds consist of a σ activation function and a point-by-point multiplication operation [34].

The described LSTM network layer comprises many cell groups, each of which comprises a forgetting gate, an input gate, and an output gate [35];

The forgetting gate is as follows:

(4) $$f_{t}=\sigma (W_{f}\left[h_{t-1},x_{t}\right]+b_{f})$$

where $f_{t}\;$ is the forgetting gate vector, $W_{f}$ is the parameter with training, $h_{t-1}$ is the unit at the last moment of improvement, $x_{t}$ is the homogenized input data, $b_{f}$ is the bias term of the forgetting gate, and σ is the activation function sigmoid;

The input gate is as follows:

(5) $$i_{t}=\sigma (W_{i}\left[h_{t-1},x_{t}\right]+b_{i})$$

(6) $$\tilde{c_{t}}=\tanh\left(W_{c}\left[h_{t-1},x_{t}\right]+b_{c}\right)$$

where $i_{t}$ is the input gate vector, *σ* is the activation function sigmoid; tanh is the activation function; $W_{i}$ is the parameter to be trained; $W_{c}$ is the parameter to be trained, $h_{t-1}$ is the output of the unit at the previous moment after the improvement, $b_{i}$ is the bias term; $b_{c}$ is the bias term, $\tilde{c_{t}}$ is the unit state vector of the current input, and $\;x_{t}$ is the input vector of the network at the current moment.

The output gate is as follows:

(7) $$o_{t}=\sigma (W_{o}\left[h_{t-1},x_{t}\right]+b_{o})$$

(8) $$h_{t}=o_{t}*\tanh\left(C_{t}\right)$$

where  σ  is the activation function sigmoid, $W_{o}\;$ is the parameters to be trained, $h_{t-1}\;$ is the unit output at the previous moment after improvement, $b_{o}$ is the bias term, $o_{t\;}$ is the output gate vector, $h_{t}\;$ is the unit output at this moment, $x_{t}$ is the input vector of the network at the current moment, and $C_{t}\;$ is the unit state vector at the current moment.

### 4.2. Network Structure and Parameter Setting

**Figure 7:**
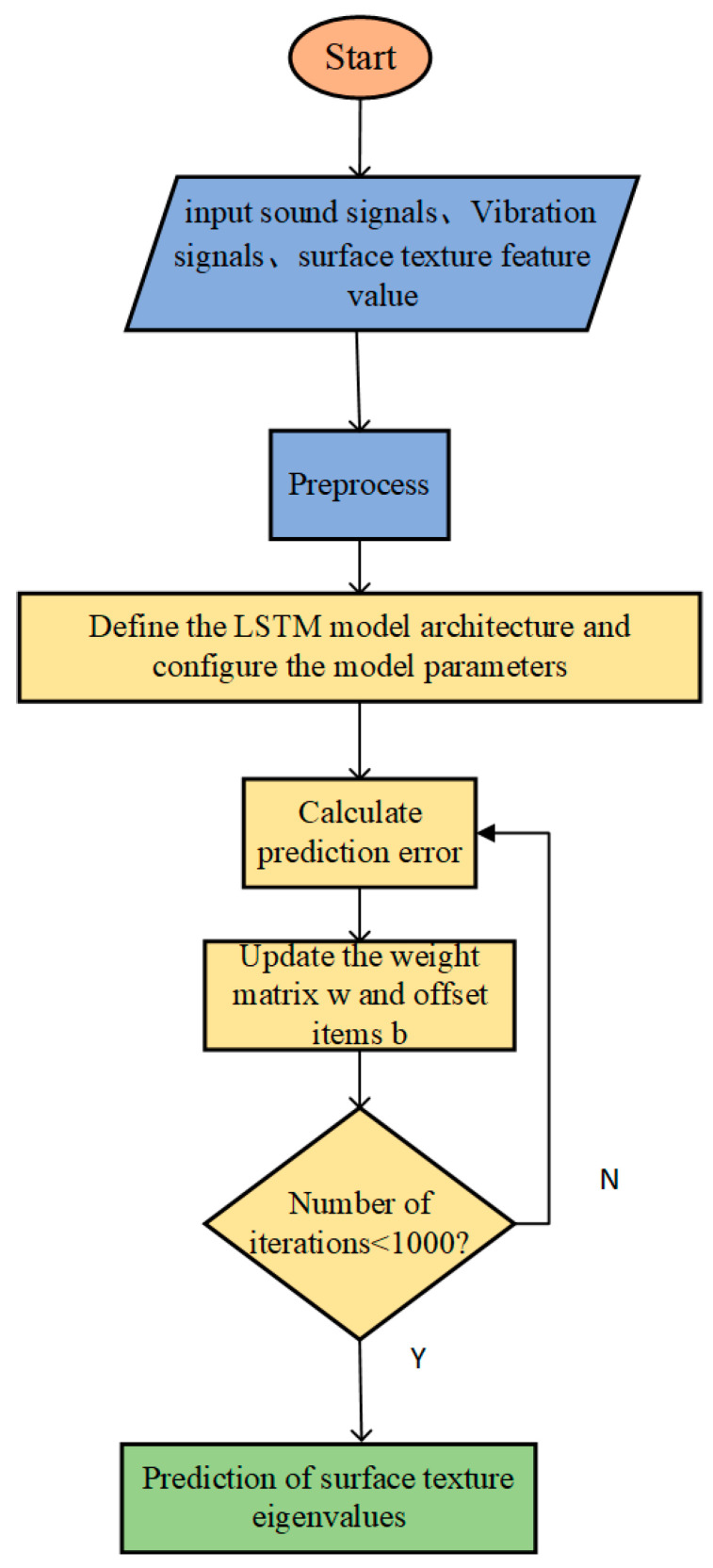
Surface texture features prediction process.

We then establish the surface texture feature value prediction model. The flowchart illustrating the prediction of the workpiece surface texture feature values using an LSTM-based approach is presented in Figure 7. Firstly, the collected signal data files are imported into the offline analysis software and converted into text format files. Secondly, the data are denoised and homogenized, 60 sets of data are taken as the training set, and 100 sets of data are taken as the test set. Third, we define the LSTM model architecture: the input dimension of the audio features is 128, the number of neural units is set to 16, the maximum number of rounds is 1000, the size of the hidden layer is 64, the batch size is 8, the MSE loss function is selected, and the learning rate is 0.001, Fourth, train the data, calculate the prediction error, and update the weight matrix W and the bias term b. MSE stands for mean squared error, which is a commonly used metric for evaluating the performance of regression models [36]. It is used to measure the square of the average difference between the predicted and true values.

(9) $$\;MSE=\frac{1}{n}\sum_{k=1}^{n}{\left(y_{k}-y_{k}^{\prime }\right)}^{2}$$

where $y_{k}$ is the kth predicted value and $y_{k}^{\prime }$ is the kth true value.

**Figure 8:**
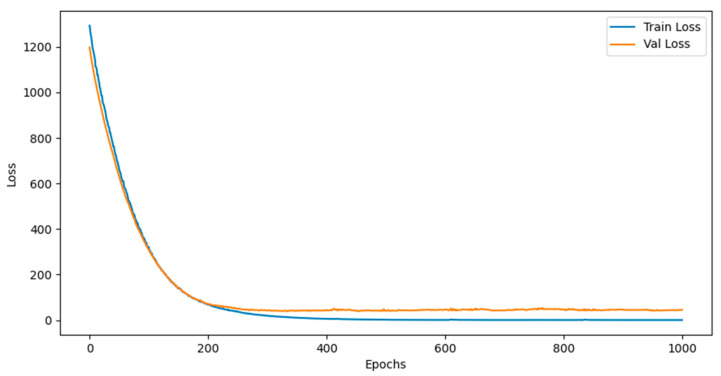
Loss function under mean square error.

After nearly 1000 iterations, the mean square error tends to converge as shown in Figure 8, and finally, the trained model is applied to realize the prediction of surface texture feature values.

### 4.3. Surface Quality Evaluation and Result Discussion 

**Figure 9:**
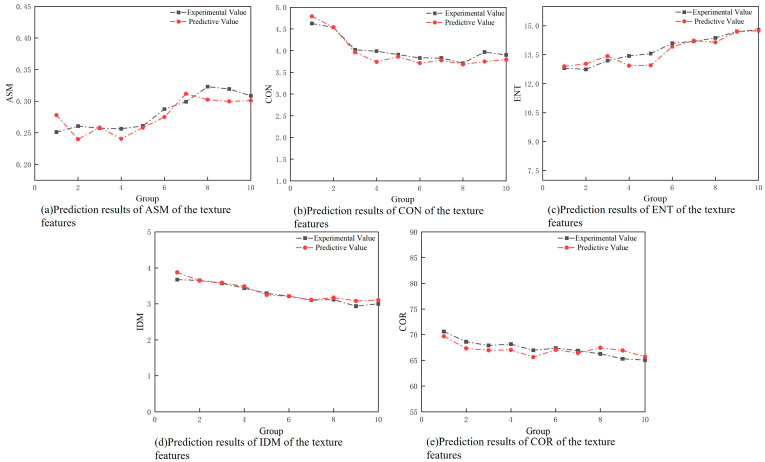
Variation in texture features with the number of cuts.

The trained model is employed to predict surface texture feature values. By comparing the graph of predicted texture features with exact texture features, as illustrated in Figure 9, it is evident that the predicted values closely align with the actual values. This indicates that the model established for predicting surface texture feature values is relatively accurate. The model’s mean square error (MSE) is 0.1645, and the mean absolute error (MAE) is 0.4275. These low error values suggest that the model can reliably predict the texture features, providing a high degree of precision in its outputs. The consistency between predicted and actual values demonstrates the effectiveness of using a long short-term memory (LSTM) neural network for this type of predictive modeling.

Following the prediction of the surface texture feature values of the machined workpiece, an evaluation of the workpiece surface quality was conducted. In Table 4, a value of 1 denotes an acceptable surface quality, whereas a value of 0 denotes unacceptable quality.

By comparing the grayscale co-occurrence matrix texture eigenvalues of the standard parts, it was observed that the accuracy rate of the model reached approximately 90% across 160 data sets. In the 160 sets of industrial field data we collected, it can be observed that the surface quality gradually deteriorates with the increase in cutting times. Because the collected data themselves have a strong correlation, the effect of using the LSTM model to evaluate the surface quality is very significant. The research results show that the LSTM model performs well in processing data with time-series changes, and it is especially suitable for such surface quality assessment tasks with significant time dependence.

Compared with studies that only consider vibration signals or sound signals, our method provides a significant advantage by integrating these two types of data. The existing prediction models using a single signal usually have large errors. For example, singular spectrum analysis (SSA) and wavelet packet transform (WPT) are used to extract vibration signal features to monitor surface roughness [37], and wavelet packet decomposition is used to decompose sound signal features to evaluate tool wear [38]. The above method only considers the characteristics of a single signal for prediction, so the accuracy of the prediction results is usually maintained at about 85%.

**Figure 10:**
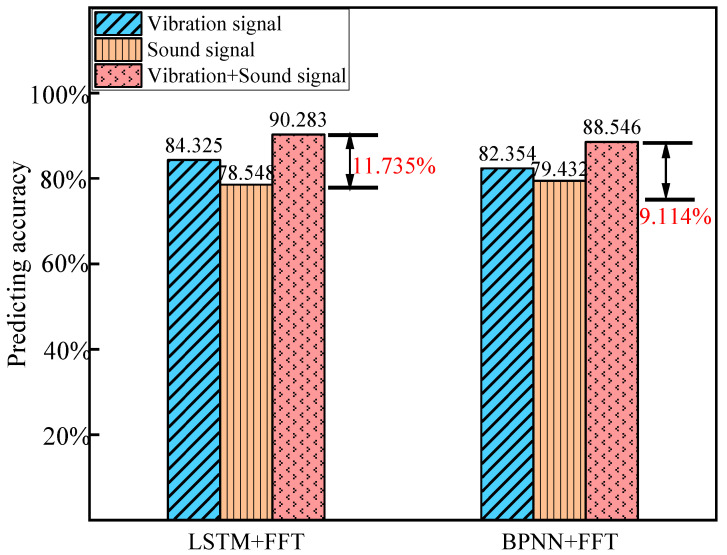
Comparison of evaluation results of models under different input signals.

In the case of using the same data feature as the model input, the prediction results of different models are compared. For the BP neural network, the activation function is set to ReLU (Rectified Linear Unit), the learning rate is set to 0.001, and the number of neurons in each layer is 50, 20, and 10, respectively. Figure 10 demonstrates that the model incorporating fused acoustic and vibration signal features achieves the highest prediction accuracy compared to models using single signal features. Furthermore, under identical input conditions, long short-term memory (LSTM) networks exhibit a superior performance, particularly for modeling data with temporal dependencies. Our method can better identify subtle changes in surface quality by utilizing the complementary characteristics of vibration and sound signals, which may be ignored in single-mode models. This ensemble method significantly improves the overall prediction performance.

The prediction model developed in this study and the proposed surface quality evaluation method is unaffected by external factors such as lighting conditions, cutting fluids, and debris, thereby reducing the time required for surface quality assessment. Additionally, this study provides a robust foundation for subsequent research in the domain of surface quality evaluation.

## 5. Conclusions

To address the complex issue of evaluating surface quality in the milling of blade-root grooves, we proposed an improved evaluation method. In this study, we collected 160 sets of industrial field data and conducted an in-depth analysis of these data. The data include vibration signals from both the spindle and the workpiece table, acoustic signals during the machining process, and post-milling surface images of the workpiece. By employing a combination of wavelet denoising and polynomial smoothing denoising techniques, smooth and clean feature representations were obtained. Correlation analysis identified that texture features exhibit a strong correlation with average amplitude, maximum amplitude, skewness, and root mean square values. These highly correlated features served as inputs for model prediction, with texture features as outputs, utilizing a long short-term memory (LSTM) network. The predictive model achieved a mean square error (MSE) of 0.1645 and a mean absolute error (MAE) of 0.4275. Furthermore, the accuracy of predicting the workpiece surface quality based on the predicted texture features was determined to be 90%. Compared with other models, it can be found that the model established by using an LSTM neural network with an acoustic vibration signal as its input has the highest accuracy. These findings underscore the efficacy of the developed workpiece surface texture feature prediction model. This research not only realizes the prediction of workpiece texture features and surface quality evaluation but also lays a solid foundation for future studies on surface quality.

In future research, we can consider the fusion of various signal characteristics under different process parameters to establish a more accurate prediction model to realize the evaluation and prediction of surface quality. At the same time, the prediction model should be further optimized to improve its mobility and applicability in different application scenarios.

## Figures and Tables

**Table 1:** Mechanical and physical properties.

**Tensile Strength** σb **(MPa)**	Yield Strength (MP)	Elongation (%)	Sectional Shrinkage ψ (%)	Work of Impact (AKV/j)	Anneal HB
**≥635**	≥440	δs≥20	≥50	≥63	≤223

**Table 2:** Information collection form.

Workpiece Materials	Spindle Speed	Cutting Speed	Feed	Milling Cutter Diameter	Milling Cutter Materials
21Cr13	350 rpm	137.7 mm/min	0.078 mm	125 mm	Cemented carbide

**Table 3:** Correlation analysis.

Texture Image Features	Most Correlate Vibration Signal Features	Most Correlate Sound Signal Features	Correlation Coefficient with Vibration Signal	Correlation Coefficient with Sound Signal
AVE_ASM	${AVE\_IMF}_{1}$	Skewness	0.882	0.904
AVE_CON	${MAX\_IMF}_{1}$	RMS	0.924	0.950
AVE_ENT	${AVE\_IMF}_{1}$	Skewness	0.884	0.898
AVE_IDM	${AVE\_IMF}_{1}$	RMS	0.893	0.896
AVE_COR	${MAX\_IMF}_{1}$	RMS	0.948	0.932

**Table 4:** Surface quality evaluation results.

No	Name	ASM	CON	ENT	IDM	COR	Surface Quality
1	Predictive Value	0.2781	4.7901	12.8995	3.8747	69.6736	1
	Experimental Value	0.2511	4.6264	12.8026	3.6728	70.62	
2	Predictive Value	0.2398	4.5409	13.0305	3.6454	67.3391	1
	Experimental Value	0.2606	4.5345	12.7417	3.6533	68.6107	
3	Predictive Value	0.2583	3.9633	13.4303	3.5864	66.9725	1
	Experimental Value	0.2573	4.0201	13.1948	3.5708	67.9129	
4	Predictive Value	0.2406	3.7427	12.9331	3.4863	67.0368	1
	Experimental Value	0.2565	3.9882	13.4364	3.438	68.1714	
5	Predictive Value	0.3218	2.5488	11.9048	3.2482	58.4772	0
	Experimental Value	0.3234	2.7415	11.9697	3.2739	58.6743	
6	Predictive Value	0.2751	3.7142	13.9174	3.2117	67.0527	1
	Experimental Value	0.2875	3.8327	14.0919	3.2144	67.3678	
7	Predictive Value	0.3119	3.7786	14.2259	3.1109	66.4437	1
	Experimental Value	0.2992	3.8315	14.1941	3.1029	66.8836	
8	Predictive Value	0.3025	3.6839	14.1422	3.1737	67.4613	1
	Experimental Value	0.2992	3.8315	14.1941	3.1029	66.8836	
9	Predictive Value	0.2997	3.7508	14.6923	3.0815	66.9251	0
	Experimental Value	0.323	3.7122	14.3677	3.1175	66.2705	

## Data Availability

Raw data are available on request.

## References

[1] Puspitasari P., Andoko A., Kurniawan P. (2021). Failure analysis of a gas turbine blade: A review. IOP Conf. Ser. Mater. Sci. Eng..

[2] Podsiadlo P., Wolski M., Stachowiak G.W. (2019). Directional Signatures of Surface Texture. Tribol. Lett..

[3] Otsuki T., Okita K., Sasahara H. (2022). Evaluating surface quality by luminance and surface roughness. Precis. Eng..

[4] Deng Y., Tan Y., Wu X., Zhu J. (2022). Local tolerance and quality evaluation for optical surfaces. Optica.

[5] Yu S., Zhao G., Li C., Xu S., Zheng Z. (2021). Prediction models for energy consumption and surface quality in stainless steel milling. Int. J. Adv. Manuf. Technol..

[6] Zhao B., Chen F., Jia X.-F., Zhao C.-Y., Wang X.-B. (2017). Surface quality prediction model of nano-composite ceramics in ultrasonic vibration-assisted ELID mirror grinding. J. Mech. Sci. Technol..

[7] Dutta S., Pal S., Mukhopadhyay S., Sen R. (2013). Application of digital image processing in tool condition monitoring: A review. CIRP J. Manuf. Sci. Technol..

[8] Gadelmawla E.S., Al-Mufadi F.A., Al-Aboodi A.S. (2014). Calculation of the machining time of cutting tools from captured images of machined parts using image texture features. Proc. Inst. Mech. Eng. Part B J. Eng. Manuf..

[9] Grzesik W., Rech J., Żak K. (2015). Characterization of surface textures generated on hardened steel parts in high-precision machining operations. Int. J. Adv. Manuf. Technol..

[10] Grigoriev A.Y., Myshkin N. Comparing surface roughness and texture concepts. Proceedings of the 8th International Conference BALTTRIB’2015.

[11] Kim D.-S., Chang I.-C., Kim S.-W. (2002). Microscopic topographical analysis of tool vibration effects on diamond turned optical surfaces. Precis. Eng..

[12] Sun H., Gao D., Zhao Z., Tang X. (2017). An approach to in-process surface texture condition monitoring. Robot. Comput. Manuf..

[13] Lu X., Zeng F., Xv K., Zhang Y., Liang S.Y. (2024). Prediction of tool wear during micro-milling Inconel 718 based on long short-term memory network. Precis. Eng..

[14] Pimenov D.Y., Bustillo A., Wojciechowski S., Sharma V.S., Gupta M.K., Kuntoğlu M. (2023). Artificial intelligence systems for tool condition monitoring in machining: Analysis and critical review. J. Intell. Manuf..

[15] Liu X., Liu S., Li X., Zhang B., Yue C., Liang S.Y. (2021). Intelligent tool wear monitoring based on parallel residual and stacked bidirectional long short-term memory network. J. Manuf. Syst..

[16] Gao K., Zhou Z., Qin Y. (2024). Gas concentration prediction by LSTM network combined with wavelet thresholding denoising and phase space reconstruction. Heliyon.

[17] Miao H., Li C., Liu C., Wang C., Zhang X., Sun W. (2024). Machined surface prediction and reliability analysis in peripheral milling operations. Int. J. Mech. Sci..

[18] Andrews A., Manisekar K., Rex F.M.T., Sivakumar G., Narayanan M. (2023). An expert system for vibration-based surface roughness prediction using firefly algorithm and LSTM network. J. Braz. Soc. Mech. Sci. Eng..

[19] Tang K.-E., Weng C.-Y., Cheng Y.-C., Liu C.-W. (2024). Typical signal anomaly monitoring and support vector regression-based surface roughness prediction with acoustic emission signals in single-point diamond turning. J. Manuf. Process..

[20] Manjunath K., Tewary S., Khatri N. (2022). Surface roughness prediction in milling using long-short term memory modelling. Mater. Today Proc..

[21] Sajjady S., Abadi H.N.H., Amini S., Nosouhi R. (2016). Analytical and experimental study of topography of surface texture in ultrasonic vibration assisted turning. Mater. Des..

[22] Bi G., Liu S., Su S., Wang Z. (2021). Diamond Grinding Wheel Condition Monitoring Based on Acoustic Emission Signals. Sensors.

[23] He Z., Shi T., Xuan J. (2022). Milling tool wear prediction using multi-sensor feature fusion based on stacked sparse autoencoders. Measurement.

[24] Jansen M. (2012). Multiscale local polynomial smoothing in a lifted pyramid for non-equispaced data. IEEE Trans. Signal Process..

[25] Li J., Cao X., Chen R., Zhao C., Li Y., Huang X. (2023). Prediction of remaining fatigue life of metal specimens using data-driven method based on acoustic emission signal. Appl. Acoust..

[26] Browne M., Mayer N., Cutmore T. (2007). A multiscale polynomial filter for adaptive smoothing. Digit. Signal Process..

[27] Dutta S., Pal S.K., Sen R. (2016). On-machine tool prediction of flank wear from machined surface images using texture analyses and support vector regression. Precis. Eng..

[28] Su H., Chen J., Li Z., Meng H., Wang X. (2023). The fusion feature wavelet pyramid based on FCIS and GLCM for texture classification. Int. J. Mach. Learn. Cybern..

[29] Sonka M., Hlavac V., Boyle R. (2013). Image Processing, Analysis and Machine Vision.

[30] Zhang X., Li M., Huang D. (2023). Surface quality and burr characterization during drilling CFRP/Al stacks with acoustic emission monitoring. J. Manuf. Process..

[31] Bhat N.N., Dutta S., Pal S.K., Pal S. (2016). Tool condition classification in turning process using hidden Markov model based on texture analysis of machined surface images. Measurement.

[32] Dutta S., Pal S.K., Sen R. (2016). Progressive tool flank wear monitoring by applying discrete wavelet transform on turned surface images. Measurement.

[33] Menezes P.L. (2016). Surface texturing to control friction and wear for energy efficiency and sustainability. Int. J. Adv. Manuf. Technol..

[34] Kulisz M., Kłosowski G., Rymarczyk T., Hoła A., Niderla K., Sikora J. (2024). The use of the multi-sequential LSTM in electrical tomography for masonry wall moisture detection. Measurement.

[35] Huang F., Li X., Yuan C., Zhang S., Zhang J., Qiao S. (2021). Attention-Emotion-Enhanced Convolutional LSTM for Sentiment Analysis. IEEE Trans. Neural Networks Learn. Syst..

[36] Filzasavitra P., Purboyo T., Saputra R. (2019). Analysis of Steganography on PNG hnage using Least Significant Bit (LSB), Peak Signal to Noise Ratio (PSNR) and Mean Square Error (MSE). J. Eng. Appl. Sci..

[37] Plaza E.G., López P.N., González E.B. (2019). Efficiency of vibration signal feature extraction for surface finish monitoring in CNC machining. J. Manuf. Process..

[38] Liu M.-K., Tseng Y.-H., Tran M.-Q. (2019). Tool wear monitoring and prediction based on sound signal. Int. J. Adv. Manuf. Technol..

